# Social determinants of health in lung cancer surgery: perspectives from family caregivers, clinicians, and healthcare system administrators

**DOI:** 10.3389/fpubh.2025.1681825

**Published:** 2026-01-15

**Authors:** Dede K. Teteh-Brooks, Madeleine Love, Aldenise P. Ewing, Betty Ferrell, Oluwatimilehin Okunowo, Audrey Shin, Loretta Erhunmwunsee, Susanne B. Montgomery, Dan Raz, Rick Kittles, Jae Y. Kim, Virginia Sun

**Affiliations:** 1 Department of Health Disparities Research, The University of Texas MD Anderson Cancer Center, Houston, TX, United States; 2 Department of Health and Human Sciences, Southern Illinois University School of Medicine, Springfield, IL, United States; 3 Division of Epidemiology, The Ohio State University, Columbus, OH, United States; 4 Division of Nursing Research and Education, Department of Population Sciences, City of Hope Comprehensive Cancer Center, Duarte, CA, United States; 5 Department of Computational and Quantitative Medicine, Division of Biostatistics, Beckman Research Institute of City of Hope, Duarte, CA, United States; 6 Department of Health Sciences, Crean College of Health and Behavioral Sciences, Chapman University, Orange, CA, United States; 7 Department of Surgery, City of Hope Comprehensive Cancer Center, Duarte, CA, United States; 8 School of Behavioral Health, Loma Linda University, Loma Linda, CA, United States; 9 Department of Community Health and Preventive Medicine, Morehouse School of Medicine, Atlanta, GA, United States

**Keywords:** electronic health record, family caregivers, lung cancer, social determinants of health, surgery

## Abstract

**Introduction:**

Social Determinants of Health (SDoH) play an integral role in health outcomes across the cancer care continuum. Despite growing recognition of SDoH in healthcare, gaps exist in systematically identifying associated social risks and needs. Insights from multiple stakeholders are necessary to optimize integration of SDoH assessments in care delivery. This study examined the impact of SDoH on lung cancer care from the perspectives of key stakeholders, including family caregivers (FCGs), providers, and healthcare system administrators, all pivotal members in care delivery and decision-making.

**Methods:**

This qualitative study was conducted at a comprehensive cancer center. Participants were interviewed regarding the integration of SDoH assessments into cancer care. Conventional content analysis was used to generate codes and themes from these key informant interviews. SDoH information were collected using the Protocol for Responding to and Assessing Patients’ Assets, Risks, and Experiences (PRAPARE) survey. Descriptive statistics were conducted for FCGs, and data were summarized using counts and percentages.

**Results:**

FCGs (*n* = 13) were predominantly non-Hispanic White females ages 60–68; other participants were five providers, (a nurse practitioner, three thoracic surgeons), and two administrators. Six themes and 19 sub-themes emerged. Participants had diverse knowledge across SDoH domains and suggested a variety of strategies for reducing caregiving burden. Three themes informed provider and administrator perspectives on the importance of integrating SDoH assessments into care delivery, with electronic health record (EHR) systems using interdisciplinary approaches that include FCG perspectives. Integrating SDoH into routine care was regarded as particularly complex in the context of lung cancer surgery, where the clinical focus is mostly on patient survival.

**Discussion:**

Addressing SDoH in cancer care delivery is complex yet essential. Systematic documentation, leveraging EHR systems, and interdisciplinary workflows are necessary to advance these efforts.

## 1 Introduction

Social determinants of health (SDoH) are the conditions of the environments in which people are born, live, learn, work, play, worship, and age that impact a broad range of health, functioning, and quality of life (QOL) outcomes (1). SDoH are categorized into five core domains, including economic stability, education access and quality, healthcare access and quality, neighborhood and built environment, and social and community context (2, 3). Social risks are adverse SDoH conditions shown to be associated with poor health outcomes and health related social needs (HRSN) are identified as unmet social risks that affect a patient’s health and well-being and may require immediate assistance. Assessing risks and addressing HRSN is essential for improving QOL for survivors and their families.

SDoH factors play a critical role in influencing patient outcomes across the cancer continuum, and lung cancer is no exception. Despite advancements in treatment and screening, lung cancer remains one of the most common and deadly cancers in the U. S. (4). The burden of lung cancer disproportionately affects individuals who experience adverse social determinants and/or HRSN (5, 6). Specifically, factors such as socioeconomic status, access to healthcare, environmental exposures, and social support systems can significantly impact the treatment and survival rates of lung cancer patients and secondarily their family caregivers (2, 7).

The unique perspectives of patients provide valuable insights on the lived experiences of individuals with a lung cancer diagnosis (7, 8). This includes the influence of SDoH factors on QOL-related issues such as financial strain, limited access to high quality healthcare, and the psychological burden associated with survivorship (7). Beyond patient perspectives, the perspectives of family caregivers (FCGs), clinicians, and healthcare system administrators are vital to understanding the full spectrum of SDoH as it relates to lung cancer care. FCGs, clinicians, and administrators play pivotal roles in cancer care delivery. Existing literature indicates that FCGs often undergo significant stress and financial challenges, impacting their potential ability to support patients (1). Meanwhile, healthcare providers and administrators face systemic challenges such as resource limitations and policy constraints, impacting their capacity to support HRSN (9, 10).

While there is growing acknowledgment of the significance of SDoH in healthcare, oncology, and beyond, gaps still exist in our understanding of SDoH, social risk, and HRSN assessment practices in lung cancer surgery. As most perspectives on HRSN are from primary care, community health centers or emergency setting (11–13), the need for additional perspectives from oncology stakeholders is warranted. Furthermore, many studies in oncology settings focus on isolated determinants or lack a comprehensive approach integrating multiple SDoH factors.

This study aimed to explore the impact of SDoH across five domains on lung cancer care from the perspectives of multiple key stakeholders, including FCGs, clinicians, and healthcare system administrators. By employing this approach, we aimed to provide insights into how such SDoH influence lung cancer care delivery and may help inform the development of targeted interventions to improve quality of care while addressing HRSN.

## 2 Materials and methods

### 2.1 Intervention, sample, and setting

This study was part of a randomized trial for FCGs and patients undergoing surgery at a National Cancer Institute designated comprehensive cancer center in Southern California. The parent study was a dyadic multimedia self-management intervention to support patients and FCGs for lung cancer surgery (14). The five-year single site trial was nurse-led and included self-management skills for FCGs pre- and post-surgery. The main outcomes of the study included psychological distress, QOL, caregiving preparedness and burden, as well as health care resources.

### 2.2 Procedures

The procedures for the study on FCGs are similar for patients and have been previously published (7). Family caregivers enrolled in the parent study participated in qualitative interviews 3 months post patient discharge. Eligibility included participants who were (a) age 21 years or older; and (b) able to read, speak, or understand English. Key informant interviews with FCGs were conducted by DT-B and co-facilitator VS using a semi-structured interview guide (Appendix A). The interview guide was developed by the investigators and pilot tested with FCGs in the same data pool. The questions were developed in three phases through review of a video, and SDoH awareness questions by the five domains related to FCG role (1). Interviews lasted between 45 and 60 min and were conducted via Microsoft Teams allowing automatic transcription of the interviews. Demographic and SDoH information were collected at baseline of the parent study using the Protocol for Responding to and Assessing Patients’ Assets, Risks, and Experiences (PRAPARE) survey (15).

Most providers and administrators were recruited from the study location. One provider was retired from the institution and recruited by senior investigators of the parent study. The lead author (DT-B) recruited and scheduled interviews with provider and administrator participants via email. Key informant interviews lasted 60 min and were completed by DT-B and co-facilitator VS. Similar to the FCG procedures, a semi-structured interview guide (Appendix B) was co-developed by the investigators and pilot tested with participants from the same pool. Questions included (1) overview of participants current or former role at their institution, (2) discussion on concepts of SDoH (3) the role of SDoH in healthcare delivery for patients, FCGs, and providers/administrators and (4) challenges and opportunities for integrating SDoH in healthcare delivery. The provider and administrator interviews focused on solution-oriented perspectives on the integration of SDoH into healthcare practice and less on knowledge of SDoH organized by the five domains. The study site’s Institutional Review Board (IRB) approved the study protocol (#17238) and procedures.

### 2.3 Data analysis

Conventional content analysis was used to develop themes of FCG experiences and providers/administrators’ perspectives about the role of SDoH in healthcare delivery. DT-B and VS collaboratively developed two codebooks for FCGs and provider/administrator analyses. The FCG codebook was organized by SDoH domains mirroring the study questions procedures. The codebooks were used by DT-B and VS in the coding of 2 transcripts. The codebooks were than revised and used to code the remaining study transcripts. Codebooks included information on SDoH domains, codes, description of codes, and examples from the initial transcripts coded by DT-B and VS. Deductive and inductive approaches were used in the data analysis process (16). Codes and themes were derived from participant interviews, as well as research on SDoH, and subject matter expert validation of data interpretation (1, 7, 8). The process for FCGs included five independent coders (DT-B, VS, JK, BF, and ML), and four reviewers (DTB, VS, JK, ML) who developed the initial themes. The process for providers/administrators involved four independent coders (DT-B, VS, ML, AS) and three reviewers (VS, ML, AS) who developed the initial themes. DT-B and VS reviewed and finalized the themes for both FCGs and providers/administrators. Any disagreements during the coding and thematic process were reviewed, discussed, and resolved by DT-B and VS along with inclusion of co-authors when applicable. Inductive thematic saturation was reached during coding and thematic analysis when no new information was found in the data (16). Member checking of provider/administrator results occurred during the analysis and manuscript development process as three of the co-authors were participants in the study (17). Descriptive statistics for FCGs were performed and demographic information were reported as counts and percentages.

## 3 Results

### 3.1 Family caregivers’ themes and sub-themes by SDOH domains

FCGs were between 60 and 68 years of age and predominantly non-Hispanic White females with no reported challenges across the five SDoH domains (Table 1). A summary of themes (6) and sub-themes (18) for FCGs (Table 2) across the five SDoH domains reflect a diversity in knowledge across SDoH factors, economic stability nuances rooted in generational acuity, and varying strategies for reducing caregiving burden.

**Table 1:** Family caregivers’ demographic characteristics by social determinants of health (SDoH) domains (*N* = 13).

SDoH domain	Characteristics	n (%)
Social and community context	Age in years, median (Q1-Q3)	62 (60–68)
	Sex assigned at birth	
	Female	7 (53.8%)
	Male	6 (46.2%)
	Ethnicity	
	Hispanic	1 (7.7%)
	Non-Hispanic	11 (84.6%)
	Choose not to answer	1 (7.7%)
	Race	
	Asian	1 (7.7%)
	Black/African American	1 (7.7%)
	White or Caucasian	9 (69.2%)
	Other^a^	1 (7.7%)
	Choose not to answer	1 (7.7%)
	Religious affiliation	
	Protestant^b^	2 (15.4%)
	Catholic	5 (38.5%)
	Jewish	1 (7.7%)
	Other	3 (23.1%)
	No religious affiliation	2 (15.4)
	Household size	
	Less than two	7 (53.8%)
	Two	2 (15.4%)
	More than two	4 (30.8%)
	Social support communication	
	1 or 2 times a week	5 (38.5%)
	3 to 5 times a week	1 (7.7%)
	5 or more times a week	6 (46.2%)
	Choose not to answer	1 (7.7%)
	Stress levels	
	Not at all	2 (15.4%)
	A little bit	4 (30.8%)
	Somewhat	2 (15.4%)
	Quite a bit	3 (23.1%)
	Very much	2 (15.4%)
	Imprisonment in the past year	
	Yes	0 (0.00%)
	No	13 (100%)
	Refugee status	
	Yes	0 (0.00%)
	No	13 (100%)
	Domestic violence	
	Yes	0 (0.00%)
	No	12 (92.3%)
	No partner in the past year	1 (7.7%)
Education access and quality	Language	
	English	13 (100%)
	Other than English	0 (0.00%)
	Choose not to answer	0 (0.00%)
	Education	
	< High school degree	0 (0.00%)
	High school diploma or GED	2 (15.4%)
	More than high school	11 (84.6%)
	Choose not to answer	0 (0.00%)
Healthcare access and quality	Main insurance	
	Medicaid/CHIP Medicaid	0 (0.00%)
	Medicare	6 (46.2%)
	Private Insurance	7 (53.8%)
	Other Public Insurance	0 (0.00%)
	Lack of access to medicine or healthcare	
	Yes	2 (15.4%)
	No	11 (84.6%)
Neighborhood and built environment	Housing situation	
	I have housing	13 (100%)
	I do not have housing	0 (0.00%)
	Choose not to answer	0 (0.00%)
	Worry about losing housing (n = 12)	
	Yes	0 (0.00%)
	No	12 (100%)
	Choose not to answer	0 (0.00%)
	Transportation access consequences	
	Medical appointments or medications	0 (0.00%)
	Non-medical meetings, etc.	0 (0.00%)
	No	13 (100%)
	Choose not to answer	0 (0.00%)
	Physical and emotional safety	
	Yes	12 (92.3%)
	No	0 (0.00%)
	Unsure	1 (7.7%)
Economic stability	Employment status	
	Unemployed	0 (0.00%)
	Full-time	8 (61.5%)
	Unemployed but not seeking^c^	5 (38.5%)
	Annual household income	
	$15,000 to $30,000	0 (0.00%)
	$30,001 to $50,000	1 (8.3%)
	$50,001 to $75,000	3 (25.0%)
	$75,001 to $100,000	3 (25.0%)
	Greater than $100,000	5 (41.7%)
	Migrant farm worker	
	Yes	0 (0.00%)
	No	12 (100%)
	Choose not to answer	0 (0.00%)
	Discharged from armed forces	
	Yes	0 (0.00%)
	No	12 (100%)
	Lack of access to food	
	Yes	0 (0.00%)
	No	13 (100%)
	Lack of access to clothing	
	Yes	0 (0.00%)
	No	13 (100%)
	Lack of access to utilities	
	Yes	0 (0.00%)
	No	13 (100%)
	Lack of access to childcare	
	Yes	0 (0.00%)
	No	13 (100%)
	Lack of access to phone	
	Yes	0 (0.00%)
	No	13 (100%)

**Table 2:** Summary of primary themes and sub-themes for family caregivers by social determinants of health (SDoH) domains.

Social determinants of health (SDoH) domain	Primary themes	Sub-themes
Not applicable	Knowledge about SDoH Factors and Integration of SDoH Information into Healthcare	Acknowledgment of social determinants of health impact on health outcomes. Integration of SDoH information into healthcare.
Economic Stability	Caregiving burden minimized by economic mobility opportunities	None or no immediate economic stability concerns. Caregiving burden experienced by the “sandwich generation” reduced by social support. Caregiving burden impact by employment status, income, and healthcare access.
Education access and quality	Degree attainment, supplemental education, and lived experience impact QOL of lung cancer surgery caregivers	Salutatory impact of education on QOL. Supplemental education to support caregiving role. Non-educational predictors of QOL.
Healthcare access and quality	Navigating the healthcare and cancer care delivery systems	Switching insurance to have more access in cancer care. Bypassing referral systems to receive timely care. Empowerment, advocacy, and persistence. Accessing quality information on lung cancer.
Neighborhood and built environment	Proximity to healthcare facilities and neighborhood environmental factors impact caregiving role	Caregiver burden of transportation & distance to medical care. Concern about environmental exposure causing cancer. Importance of neighborhood and built environment for ease of caregiving.
Social and community context	Availability of social support and caregiving coping strategies	Availability of social support. Shifts to caregiver roles and responsibilities. Caregiver coping strategies. Use of religion/spirituality as coping strategy.

### 3.2 Theme 1: Knowledge about SDoH factors and integration of SDoH information into healthcare system

#### 3.2.1 Acknowledgment of SDoH impact on health outcomes

While some FCGs were familiar with the concept of SDoH and its potential health impact, many individuals were new to this concept. Without such prior knowledge about SDoH, most FCGs acknowledged however that conceptually an individual’s unique combination of environment, education, economic situation, social context, and health care access could have either positive or negative implications on health outcomes. After the discussion, some FCGs came to a deeper understanding of the ways in which social factors could impact health outcomes.

#### 3.2.2 Integration of SDoH information into healthcare

Overall, FCGs expressed positive sentiments about adoption of SDoH information into the healthcare setting while others felt its value was limited as there was not much in one’s situation that could be changed at the point of diagnosis. Nonetheless, FCGs recognize the importance of the care team knowing about the life circumstances of the patients including if there was adequate support when not in their immediate care. The SDoH information integration into the healthcare setting was seen as beneficial under certain circumstances, such as if the patient’s situation required support for various HRSN. For example, it could be potentially valuable for a patient who must travel a great distance, has no family close by, or has limited funds. Therefore, the utility of including SDoH information in the healthcare delivery practice should include tangible solutions and not collected for merely informational purposes.

### 3.3 Theme 2: Caregiving burden minimized by economic mobility opportunities

The economic stability domain is related to income accessibility and mobility barriers or opportunities that may or may not allow individuals to meet their health needs. Three sub-themes within this domain included *none or no immediate economic stability concerns*, *caregiving burden experienced by the “sandwich generation” reduced by social support availability*, and the *impact of retirement, income, and healthcare experience on caregiving burden*. The FCGs of lung cancer surgery patients in our study worked full time (8%), were nearing retirement, or were retired (39%). Nearly half of FCGs (42%) also reported an annual income of greater than $100,000 with no food, clothing, or utility uncertainties. Participants also had access to savings, stocks, and stable living conditions (e.g., homeowners). While most participants did not report economic stability challenges, one expressed distress about financial security after retirement and the desire to “live comfortably.” Moreover, a participant with dual caregiving roles provided support to a parent (lung cancer patient) as well as their husband and children. Despite the increase in caregiving responsibilities, they reported a less burdensome experience because of the support received from their spouse. Additionally, the transition to retirement, support provided by the healthcare team, and lack of worry about income may have reduced the potential economic and emotional burden of caregiving. Nevertheless, one participant acknowledged that the impact of employment status, income, and access to healthcare experienced by cancer patients is not equitable.

### 3.4 Theme 3: Degree attainment, supplemental education, and lived experience impact QOL of caregivers

Caregivers agreed with the premise that education is a prerequisite for achieving a better quality of life and the impacts of health are salutatory. There were, however, divergent views on the definition and type of education needed to achieve the desired health outcomes. Moreover, 85% of caregivers had more than a high school diploma. Degrees ranged from an associate in ornamental horticulture, to a Bachelor of Science, a master’s in business administration and Doctor of Medicine (e.g., OBGYN provider). There were three sub-themes on the salutatory role of education, supplemental education to support caregiving role, and non-educational predictors of QOL for lung cancer surgery caregivers.

#### 3.4.1 Salutatory impact of education on QOL

Most caregivers agreed with the statement that higher levels of education equate to a healthier, longer life and improves an individual’s QOL. For instance, education may provide knowledge on environment-health interactions, healthy nutritional habits at an early age, economic mobility opportunities, health literacy, and healthcare access and navigation. Additionally, completing a degree program may provide baseline knowledge on health, and supplemental education may be required to adequately prepare caregivers for their roles. The surgery preparation materials including expectation before and after surgery, crisis management, and prior caregiving or healthcare experiences were noted as foundational content for caregivers.

#### 3.4.2 Supplemental education to support the caregiving role

Furthermore, some caregivers provided additional narratives about levels of education through degree attainment and QOL. For example, a degree would not determine your QOL rather other impactful factors may include having “street smarts,” living a healthy life, and availability of social support. One caregiver alluded to the negative consequences of achieving higher levels of education, including job related stressors that may diminish QOL.

### 3.5 Theme 4: Navigating healthcare and cancer care delivery systems

For FCGs of persons faced with lung cancer surgery, the interactions and experiences with the cancer care delivery system varied and largely portrayed the experience by FCGs navigating the cancer care delivery system along with the patients. Their interactions with the cancer care delivery system included driving patients to their appointments, as well as participating in clinical visits with the healthcare team. Some FCGs had very minimal contact with the cancer care delivery system (e.g., primarily driving patients to appointments) while others were heavily involved beginning with the diagnosis phase. Additionally, FCGs themselves had no access challenges for their own healthcare needs; several had their own cancer diagnoses and were being followed for surveillance. A dual diagnosis, particularly when the FCG and patient were diagnosed around the same time, created a mutual understanding and a shared perspective on each other’s survivorship journeys. From this context, four sub-themes described their navigation of the healthcare and cancer care delivery systems.

#### 3.5.1 Switching insurance to have more access in cancer care

Navigation of the system for some involved switching insurance to access the desired cancer center and cancer care delivery system. Overall, in this group of FCGs, there were minimal to no insurance-related access barriers. Having certain types of insurance, particularly HMOs, were noted by FCGs as a potential access barrier, limiting patients to a specific network of oncologists and restricting their choices in care delivery systems, ultimately impacting access to quality cancer care.

#### 3.5.2 Bypassing referral systems to receive timely care

To ensure a timely diagnosis and treatment, FCGs may bypass the standard referral systems by directly contacting cancer centers to secure appropriate care for patients. Bypassing the standard referral system led to higher deductibles and additional out of pocket costs to access the preferred cancer care delivery system in a timely manner.

#### 3.5.3 Empowerment, advocacy, and persistence

FCGs often served as the advocate for patients during direct contact with providers. An important part of navigating cancer care delivery systems is empowering patients and families to take control of their healthcare journey, including understanding and considering their options. While empowerment, persistence and advocacy are important, FCGs also recognized that those without access challenges or economic stability often have more options than those facing significant access barriers.

#### 3.5.4 Accessing quality information on lung cancer

Though most FCGs were satisfied with the treatment information provided, some expressed a need for more detailed information specifically about lung cancer as a disease. FCGs frequently used the internet for information on lung cancer; however, this approach often presented a paradox, as it exposed FCGs and patients to both reliable and potentially misleading information. Some of this information, when presented without context, can be deeply distressing for FCGs and patients. Additional support and knowledge were perceived as helpful, particularly during challenging times. Timely responses from the oncology care team were also seen as essential to quality cancer care, particularly during the weekend when unanticipated challenges arose.

### 3.6 Theme 5: Proximity to healthcare facilities and neighborhood environmental factors impact caregiving role

Caregivers recognized the importance of neighborhood and built environment for their caregiving and overall health. Deciding to access their preferred cancer center led some to travel between 10 min to 3 h to receive care. Some patients reduced travel time for certain clinic visits, like infusions, by going to nearby medical centers, and reserving longer trips for survivorship care visits at their preferred cancer center.

#### 3.6.1 Caregiver burden of transportation and distance to medical care

For patients living further away from medical care, there was a greater burden placed on FCGs who provided transportation. The demands included having to manage patient symptoms during transportation. FCGs time demands include the treatment time and roundtrip transportation time. One FCG felt information and support was lacking from providers who did not fully recognize the burdens of travel. By the same token, FCGs who lived close to the patient and close to medical care appreciated this convenience.

#### 3.6.2 Concern about environmental exposure causing cancer

Many FCGs raised questions about the role of environmental exposures in possibly contributing to the development of cancer. Multiple FCGs speculated about the role of air pollution in the etiology of lung cancer. Other FCGs noted pollution in ground water or from oil spills. These environmental factors were raised particularly with FCGs of patients who never smoked. There was also recognition that more privileged neighborhoods, closer to beaches, may be more desirable due to better air quality.

#### 3.6.3 Importance of neighborhood and built environment for ease of caregiving

Many caregivers reported that they were fortunate to live in “good” neighborhoods. The positive attributes of these neighborhoods included access to outdoor spaces, safety, access to healthy food options, and proximity to healthcare. Access to outdoor space, neighborhood safety, and air quality were all factors that impacted FCGs ability to spend time outdoors. FCGs noted that being in a healthy environment supported their caregiving role. Many caregivers who felt they lived in healthy neighborhoods were aware of other nearby areas they felt were less healthy. Characteristics of these neighborhoods included traffic, proximity to freeways, crime, homelessness, and abundance of fast food. Some FCGs reported that their own neighborhoods and built environments were different than the patients.

### 3.7 Theme 6: Availability of social support and caregiving coping strategies

#### 3.7.1 Availability of social support

For many FCGs, social support was readily available by way of children, siblings, spouses, friends, and neighbors. One caregiver served as the patient’s sole source of social support. Social support was demonstrated to patients and FCGs in the form of bringing over food, in-person visits, providing rides to and from appointments, church prayer groups, phone calls, and supportive text-messages. In some cases, COVID-19 presented challenges in terms of face-to-face socialization, but relationships were still maintained through phone calls and video chats. Throughout the caregiving journey, many FCGs’ relationships grew stronger with both the patient and family members while in some cases relationships stayed the same compared to before the caregiving experience.

#### 3.7.2 Shifts to caregiver roles and responsibilities

Many caregivers had to adapt to changes in roles and responsibilities throughout the caregiving journey. Most caregivers reported providing emotional support to the patient by staying “strong” and serving as a cheerleader throughout the treatment process. Symptom management was a major shift in responsibilities for many by performing tasks such as medication management, changing bandages, flushing ports, sterilizing wounds, tending to dietary and hydration needs. Other changes to caregiver roles and responsibilities included adoption of more general household duties such as laundry, cleaning, and childcare.

#### 3.7.3 Caregiver coping strategies

To cope with the caregiving journey, caregivers adopted a variety of strategies. The practice of keeping a positive outlook and attitude was a commonly referenced strategy. Many caregivers turned to various forms of physical activity, such as going to the gym, walking, hiking, and playing recreational sports. Additionally, fostering social relationships was a common coping mechanism by keeping company and doing activities with other family members, friends, and the patient. Other caregivers turned to creative outlets such as art and learning to play piano. Another caregiver found solace in informational support through searching the internet for information to be better equipped as a caregiver.

#### 3.7.4 Use of religion/spirituality as coping strategy

The majority of FCGs expressed the use of religion and spirituality to cope with their caregiving journey, leaning into their faith to provide comfort. This practice varied for many FCGs, ranging from daily prayer, meditation, going to church every Sunday, to listening to religious music. Others did not engage in an organized religion but rather practiced their own understanding of their spirituality. Only two individuals explicitly expressed no use of religion/spiritual coping methods.

### 3.8 Providers and administrator themes and sub-themes on integration of SDOH assessments into healthcare delivery practice

Providers were primarily physician scientists with expertise in thoracic surgery, nursing, familiarity with the concepts of SDoH, and QOL ranging in experience from six to over 30 years. Administrators included a public health practitioner and nurse with expertise in SDoH, QOL, and community engagement with over three decades of research and practice experience. As shown in Table 3, discussions with providers (1 nurse practitioner, 3 thoracic surgeons) and administrators (one public health and one nurse scientist) included the importance of integrating SDoH assessments into health care delivery models for patients including interdisciplinary approaches for solutions and the use of electronic health records. Providers and administrators also discussed the critical role of FCGs, and the complexities of their integration into the cancer care delivery workflow. Additionally, themes and sub-themes with example quotes from participants are shown in Appendix C, D, respectively.

**Table 3:** Overview of key themes and sub-themes for providers and administrators on incorporating social determinants of health (SDoH) assessments into healthcare delivery.

Primary themes	Sub-themes
*Theme 1*: Importance of SDoH integration into healthcare delivery models for patients.	Healthcare system reckoning on the reality of inequity and fragmentation of care. SDoH models of care that are not entirely disease focused. Sustainability of SDoH-driven models of care. Interconnectedness of SDoH and QOL
*Theme 2*: Interdisciplinary approaches to collection of SDoH data in healthcare delivery for patients.	Collection of SDoH data in healthcare delivery. Utilization of Electronic Health Records (EHRs).
*Theme 3*: The role of family caregivers (FCGs) in cancer care delivery in the context of SDOH.	Essential role of family caregivers in cancer care delivery. Integration of FCG’s SDoH information into cancer care delivery workflow.

### 3.9 Theme 1: Importance of SDoH integration into healthcare delivery models for patients

Providers and administrators unanimously agreed SDoH play a critical role in cancer care delivery. At an administration level, determinants such as food insecurity, transportation, financial toxicity, housing, and digital access are critical components to improve patient outcomes and well-being. There is acknowledgment that more work is necessary to consistently address SDoH in healthcare delivery–including the need for care delivery systems to recognize the reality of the current state of inequity and fragmentation in cancer care. Providers and administrators provided several suggestions on strategies to integrate SDoH into cancer care delivery. For instance, administrators suggested leveraging interdisciplinary models of care, moving away from a disease-only focused care model, and systematically integrating various expertise to address SDoH risk factors. Additionally, this information should be well-integrated and acted upon appropriately, mobilizing resources rather than simply collecting data. Furthermore, a practical approach should acknowledge the complexity of the healthcare system while ensuring a coordinated effort to collect SDoH information efficiently, reducing redundancy, and minimizing patient survey fatigue. Together, establishing a sustainable and effective care delivery system that addresses SDoH requires strong leadership support to develop a practical process and infrastructure.

Providers and administrators alike also acknowledged that SDoH have a profound and direct impact on patients’ QOL and health outcomes. Factors like homelessness, lack of resources, lack of insurance coverage, transportation difficulties, and limited social support were exemplified as elements that can adversely affect treatment adherence, outcomes, and overall well-being. Specifically, one administrator highlighted how the intersectionality of different converging SDoH factors can influence the way a patient experiences their course of care, thus impacting their QOL. Further, providers recognized the importance of routinely assessing SDoH to better understand and address patients’ needs beyond medical conditions. Additionally, suggestions revolved around limiting barriers to care by providing patient resources, such as support and education. One provider recommends the importance of treatment and diagnosis education for patients to have consciousness in decision-making to improve care delivery. The intimate relationship between social factors and patient experiences emphasizes the necessity of addressing SDoH to improve the QOL for individuals with cancer.

### 3.10 Theme 2: Interdisciplinary approaches to collection of SDoH data in healthcare delivery for patients

The importance of interdisciplinary collaboration was emphasized by both providers and administrators. There was a shared recognition that different professionals, such as nurses, social workers, and physicians, play a pivotal role in providing comprehensive, patient-centered care. Accordingly, collection of SDoH data in healthcare delivery should employ interdisciplinary approaches. Collaboration and information sharing across these disciplines were seen as essential for improving patient outcomes. It is also important to note while providers and administrators are committed to supporting a systematic workflow for identifying and addressing SDoH risks and needs, there remains a tension between this effort and their primary focus on treating the patient’s disease, especially for lung cancer care.

For example, an administrator and provider noted that nurses are well qualified to serve as a generalist in assessing the four dimensions of QOL, physical, psychological, social, and spiritual, while then mobilizing support services and resources when it is recognized that the patient has significant HRSN. The administrator further stated the belief that quality cancer care should require every patient to be seen by a social worker. The nurse practitioner has played a key role in connecting patients with expressed HRSN to appropriate support services or referring families to a social worker. While they agree a nurse or physician assistant can conduct social risk assessments for patients, there is less clarity regarding how often these assessments should be performed and the specific questions that should be included. Currently, there is an informal process for addressing patient needs based on the severity of the issue. This workflow involves social workers meeting patients at the clinic for follow-up and providing available resources. However, there is no established system to track whether these patients’ needs have ultimately been met.

The potential role of technology, particularly the electronic health record (EHR, e.g., *MyChart*), was discussed by providers and administrators to improve communication, streamline processes, and address gaps in care. The current model incorporates a HRSN screener into the EHR with responses collected at intake regarding financial security, housing security, food security, and transportation. However, some aspects of this model have limitations in that information is not always accurately completed or followed up upon, presenting concerns regarding effectiveness and accessibility of such solutions. One provider suggested the use of electronic kiosks to collect information at intake, as well as noting the potential challenges associated with language barriers and literacy. An administrator suggested the idea of allowing patients to update their information in MyChart, providing the option to privately reflect life changes such as a lost job. Providers and administrators agreed SDoH assessment information should be incorporated into the EHR, noting that only after such information is incorporated into the chart can it then begin to impact and inform care. Once the EHR SDoH workflow is better refined at the study institution, providers and administrators expressed optimism in its utility in addressing patient needs.

### 3.11 Theme 3: The role of family caregivers in cancer care delivery in the context of SDoH

Providers and administrators embraced FCGs as key stakeholders in the cancer care delivery process, highlighting the critical role they play in providing care to patients. Family caregivers often become the primary support system after discharge and are thus integral to the patient’s well-being. However, considering the complex healthcare environment, the FCG’s role can be overlooked. Providers and administrators emphasized that a comprehensive social assessment of a patient should include information about the FCG. Likewise, administrators underscored the importance of understanding the unique HRSN of caregivers to provide optimal care for the patient. For example, as one administrator noted, there’s an interplay between the patient’s HRSN and the caregiver’s ability to offer effective support.

Providers and administrators noted there is no current system in place for identifying social risks and documenting FCG’s HRSN beyond research. While acknowledging the value of FCG SDoH information, some providers expressed that it may be logistically difficult to collect and integrate their information into the current care delivery workflow. Given the existing challenges of limited time, energy, and resources in the healthcare setting, adding social risk and needs assessments of caregivers could complicate the process further. Additionally, providers shared concerns of documentation storage within the EHR while maintaining patient privacy if FCG assessments are collected. Identifying social risks and addressing the HRSN of patients has not yet been optimized at the current institution, therefore providers recommended an initial focus on patients’ needs toward improving QOL in the context of SDoH. Moreover, providers indicated SDoH factors are often shared between patients and caregivers, so conducting a systematic assessment of the patient may aid in efforts to identify social risks and address HRSN of both groups. Alternatively, an administrator expressed enthusiasm about the potential utility of EHR-integrated FCG information for HRSN research, noting that it could help inform the development of an effective workflow in the future.

## 4 Discussion

Social determinants of health factors influence QOL of patients and FCGs across the cancer continuum. Patients, FCGs, providers, and healthcare administrators collectively acknowledge this perspective. The integration of SDoH assessments into a cancer care delivery system where social risks are identified, and HRSN of patients and FCGs are addressed is complex. This complexity was noted by all stakeholders throughout this and previous studies (7). Recommendations also included support for an interdisciplinary care delivery system model that centers the social experiences of patients and their families while considering the capacity and limitations of the healthcare system.

Furthermore, descriptions of SDoH factors for providers and administrators included a “whole person health” framework (19), which warrants looking beyond the patient’s diagnosis and biology. Providers engaged in discussions about the patient’s personal experiences, education, and social support availability, recognizing the importance of social and environmental factors in patient outcomes. They also acknowledge a patient’s background is critical to providing optimal cancer care. Information on barriers such as access to care, education in the context of health literacy, are factors that may be relevant to improving the care experience and improving QOL of patients. Administrators’ experiences with SDoH involved understanding the social risk and HRSN of patients in the context of behavioral interventions. There were noted gaps in translation of these research based interventions into clinical practice (18).

An initial step toward identifying SDoH is documentation of these factors as part of the healthcare delivery system. There’s a lack of a systematic workflow for identifying SDoH associated risk and HRSN as well as limited intervention resources for patients and especially for their FCG at the study site. Follow-up studies should consider the adaptation of the International Classification of Diseases (ICD)-10 codes to identify social risk and HRSN (including HRSN of FCG) of thoracic surgery patients. The ICD is the system for classification of diagnoses and healthcare visits (20). The ICD-8 edition already includes social, behavioral, and economic need, assessed via V-codes that were rarely used (21). ICD-10 edition revisions, launched in 2015 expanded these codes to include additional SDoH classifications now known as “Z-codes” (Z55-Z65) related to social risk and needs of patients. The ICD-10 codes were modified in 2018 (ICD-10-CM) to include reporting by all providers including physician and other clinicians who can establish a patient’s diagnosis. These codes do not include FCG related challenges.

The uptake of use of ICD-10 codes across the healthcare system remains low but promising (22–24). There was enthusiasm from providers and administrators as the development of an EHR documentation of SDoH workflow integration was underway at the cancer center. However, it is unclear if the system will include the notation of Z-codes to systematically assess SDoH associated risk and HRSN of patients. Of note, the percentage of hospital usage of Z-codes increased from 41 to 70% between January 2016 and 2017 using the National Inpatient Sample (22). Hospitals that are likely to use these codes include larger, private not-for-profit and urban teaching hospitals. Though all patients in the study were insured (Medicare or private insurance), patients covered by Medicaid or uninsured were more likely to have documentation under ICD-10 Z-code (22, 23). Some of the cited barriers to documentation include the lack of financial incentive, lack of provider awareness and training on use, patient privacy concerns, and EHR limitations (25).

Nonetheless, improving documentation of SDoH factors in a surgical oncology settings may enhance risk stratification and patient care planning (26). For example, patients with an associated ICD-10 code had longer length of stay and higher odds of being discharged to a skilled nursing facility (27). Additionally, colorectal cancer patients with documented SDoH had 16% higher odds of experiencing 30-day postoperative complications than their counterparts without documentation (28). Without a systematic integration of SDoH factors into healthcare workflows at the cancer center, there may be gaps in care quality for patients and their families. Though, the initial focus as discussed by providers and administrators of this study are patients, there’s still a critical need to include FCGs in this integration process.

The role of FCGs was recognized by participants as integral to supporting patients throughout their survivorship journeys. The emphasis on the well-being of both patients and FCGs pose unique challenges as only the patient’s information is obtained and included in the EHRs. Providers in this context are focused on saving the lives of their patients. Administrators also recognize the important role of FCGs however their integration into the healthcare delivery infrastructure is less clear. Evidenced-based interventions to reduce the burden and psychological strain of caregiving and improve QOL of FCGs are available and some have been integrated into healthcare settings (13). For example, a cancer center in Philadelphia adapted its existing patient portal to include a patient-caregiver interface (13). Patients identified a primary caregiver, who then received login credentials to access the portal independently. The system enabled separate and joint engagement to support QOL outcomes (e.g., physical, emotional, and financial), with responses shared with the care team and referrals made to social work when caregivers reported high distress. The portal was perceived as acceptable by patients, FCGs and providers, with noted benefits to clinical care.

Additionally, the cancer support source-caregiver is a web-based distress screening and referral tool designed by the Cancer Support Community (CSC) has been used to identify caregivers at risk for depression, distress, and burnout (29). The tool has been implemented across the CSC’s national network, demonstrating effectiveness in reaching diverse populations, including rural and younger caregivers. FOCUS is an effective psychoeducational program shown to improve QOL of cancer patients and FCGs including physical and mental health benefits, reduction in caregiver burden and improved coping skills (30). The FOCUS program has been adopted for CSC (FOCUS-CSC) in Michigan, California, and Ohio with similar intervention effectiveness (31, 32). The Program for the Study of Cancer Caregivers at Memorial Sloan Kettering Cancer Center (MSKCC) was developed to provide caregivers experiencing significant burden with effective psychosocial services (33). The program leverages the four pillars of clinical care, research, professional teaching, and outreach. Housed within this comprehensive program is the Caregivers Clinic at MSKCC (34). where caregivers receive their own unique medical records. Lastly, the MSKCC has developed a multidimensional training curriculum for faculty and staff in addition to outreach activities to disseminate information and services beyond MSKCC.

Furthermore, the National Strategy to Support Family Caregivers includes over 350 federal actions with five goals in providing resources and services to FCGs with the purpose of improving QOL (35). These goals include increased awareness and outreach, partnership building, increased services and support, financial security, and expansion of research and interventions to support FCGs. The Caregiver Advise Record Enable (CARE) Act is a part of this strategy and provides support for FCGs of all ages and any health diagnosis (36, 37). The act is law in 44 states including California, which went into effect in January 2016. Like the interventions described previously, the act advises patients to identify a caregiver once hospitalized, include FCGs’ name and contact information in the patient’s medical record and notification is provided to this individual upon hospital discharge including education on medical/nursing tasks for at home care. This is important legislation that supports the engagement of FCGs of lung cancer surgery patients. However, the implementation of this legislation at the study location is unknown and the inclusion of FCGs information into electronic medical records is not a part of the current workflow for our study participants as the current EHR system is patient centered. Moreover, it is not clear how the current political reality of significant cuts to Medicare and Medicaid will further impact not only patient care and system improvement but also the needed expanded attention to FCG related systems expansions.

### 4.1 Strengths and limitations

The study was implemented at one institution focusing on cancer care, with uniform perspectives from FCGs, providers, and administrators regarding patient care and delivery in the context of SDoH influences. However, most participants were White or Caucasian (69.2%) from privileged neighborhoods (income < $100,000), educated, and with access to healthcare (84.7% insured). The participants of the study reflect the catchment area of the cancer center. The privileged status of the participants may have shaped their perceptions of SDoH; but this context was not queried during data collection. While this limited our understanding of under resourced communities, this study highlights the importance of SDoH acknowledgement and integration into patient and FCG care delivery within lung cancer care. Future studies should integrate perspectives from low-income, minoritized, uninsured, or Medicaid-covered patients and FCGs who may carry the burden of HRSN.

The results also inform our understanding of SDoH related burdens for FCGs, and the limitations of the healthcare system to support the HRSN of both patients and FCGs. For example, potential barriers to SDoH integration as part of lung cancer surgery care may include lack of time to assess/collect the information and minimal time to address SDoH related challenges preoperatively. Additionally, the role of the perioperative care team in capturing SDoH information and managing SDoH challenges need to be clarified and formalized in future studies.

Therefore, this study highlights the need for systematic recording and interdisciplinary processes to support the integration of SDoH workflows in lung cancer care. While beyond the scope of the current work, future efforts should focus on developing specific operational pathways to address non-medical needs experienced by patients and caregivers throughout their survivorship journeys. Moreover, although examples exist of FCG data integration through EHRs at similar cancer centers (31, 32, 34), current findings do not include the feasibility of integration in the context of legal constraints, EHR redesign, or Health Insurance Portability and Accountability Act (HIPAA) implications. These perspectives should be considered in the holistic implementation of future interventions for this population.

Lastly, although inter-coder reliability was not formally calculated, the use of a conventional content analysis approach guided by the SDoH framework, along with independent coding and an iterative process to refine the codebook and finalize themes helped ensure the rigor of our methods and findings.

### 4.2 Conclusion

FCGs, providers, and administrators acknowledge the importance of SDoH factors on QOL outcomes for both patients and caregivers. Integrating SDoH assessments into EHRs holds promise for advancing a ‘whole person health’ framework that supports patients’ HRSN. While FCGs are undoubtedly vital members of the care team, documentation of their SDoH-related risk factors remain limited outside of research settings. The use of Z-codes can facilitate the integration of SDoH data into clinical practice and understanding the barriers providers face in using them is critical for developing a systems-level approach to managing the risks and HRSN of patients and FCGs. However, until risks and support for HRSN are systematically integrated and prioritized (2)—‘knitted’ into the fabric of the healthcare system—discussions about SDoH in cancer care will be limited to measurements rather than interventions that advance equity for the most vulnerable.

## 5 Clinical practice implications

Understanding SDoH from the perspectives of FCGs, clinicians, and healthcare system administration can assist with initiatives to improve the quality of cancer care delivery. It reinforces the importance of building care delivery systems that are patient- and family-centered, with acknowledgement that understanding the patient/FCG’s cancer experiences should be comprehensive and inclusive of their SDoH context. Cancer care delivery systems can leverage the SDoH perspectives to prioritize resources and develop programs to support patient and FCG’s HRSN.

## Data Availability

The raw data supporting the conclusions of this article will be made available by the authors, without undue reservation.

## References

[1] Teteh DK Love M Ericson M Chan M Phillips T Toor A . Social determinants of health among family caregiver centered outcomes in lung cancer: a systematic review. J Thorac Dis. (2023) 15:2824–35. doi: 10.21037/jtd-22-1613, 37324097 PMC10267915

[2] Tucker-Seeley R Abu-Khalaf M Bona K Shastri S Johnson W Phillips J . Social determinants of health and Cancer care: an ASCO policy statement. JCO Oncol Pract. (2024) 20:621–30. doi: 10.1200/OP.23.00810, 38386945

[3] McDougall JA Hastert TA Teteh DK Rogers CR Moss JL Ochoa-Dominguez CY . Addressing social risks to accelerate health equity in cancer prevention and control. Cancer Epidemiol Biomarkers Prev. (2024) 33:337–40. doi: 10.1158/1055-9965.EPI-23-1212, 38317629 PMC12449779

[4] Kratzer TB Bandi P Freedman ND Smith RA Travis WD Jemal A . Lung cancer statistics, 2023. Cancer. (2024) 130:1330–48. doi: 10.1002/cncr.35128, 38279776

[5] Brock BA Mir H Flenaugh EL Oprea-Ilies G Singh R Singh S. Social and biological determinants in lung Cancer disparity. Cancers (Basel). (2024) 16:612. doi: 10.3390/cancers16030612, 38339362 PMC10854636

[6] Raman V Yong V Erkmen CP Tong BC. Social disparities in lung cancer risk and screening. Thorac Surg Clin. (2022) 32:23–31. doi: 10.1016/j.thorsurg.2021.09.011, 34801192

[7] Teteh DK Ferrell B Okunowo O Downie A Erhunmwunsee L Montgomery SB . Social determinants of health and lung cancer surgery: a qualitative study. Front Public Health. (2023) 11:1285419. doi: 10.3389/fpubh.2023.1285419, 38026333 PMC10644827

[8] Teteh DK Barajas J Ferrell B Zhou Z Erhunmwunsee L Raz DJ . The impact of the COVID-19 pandemic on care delivery and quality of life in lung cancer surgery. J Surg Oncol. (2022) 126:407–16. doi: 10.1002/jso.26902, 35460517 PMC9088468

[9] Siler S Mamier I Winslow BW Ferrell BR. Interprofessional perspectives on providing spiritual Care for Patients with Lung Cancer in outpatient settings. Oncol Nurs Forum. (2019) 46:49–58. doi: 10.1188/19.ONF.49-58, 30547964 PMC7008957

[10] Zettler ME Feinberg BA Jeune-Smith Y Gajra A. Impact of social determinants of health on cancer care: a survey of community oncologists. BMJ Open. (2021) 11:e049259. doi: 10.1136/bmjopen-2021-049259, 34615676 PMC8496396

[11] Assaf RR Assaf RD Padlipsky PS Young KDA. A family-centered approach to social needs awareness in the pediatric emergency department. PEC Innov. (2024) 4:100283. doi: 10.1016/j.pecinn.2024.100283, 38689830 PMC11059452

[12] Broaddus-Shea ET Jimenez-Zambrano A Holliman BD Connelly L Huebschmann AG Nederveld A. Unpacking patient perspectives on social needs screening: a mixed methods study in western Colorado primary care practices. Patient Educ Couns. (2024) 125:108298. doi: 10.1016/j.pec.2024.108298, 38735120

[13] Kreuter MW Thompson T McQueen A Garg R. Addressing social needs in health care settings: evidence, challenges, and opportunities for public health. Annu Rev Public Health. (2021) 42:329–44. doi: 10.1146/annurev-publhealth-090419-102204, 33326298 PMC8240195

[14] Sun V Raz DJ Erhunmwunsee L Ruel N Carranza J Prieto R . Improving family caregiver and patient outcomes in lung cancer surgery: study protocol for a randomized trial of the multimedia self-management (MSM) intervention. Contemp Clin Trials. (2019) 83:88–96. doi: 10.1016/j.cct.2019.07.002, 31279090 PMC6661176

[15] National Association of Community Health Centers. *Protocol for Responding to and Assessing Patients’ Assets, Risks, and Experiences (PRAPARE)*. (2016). Available online at: https://www.nachc.org/research-and-data/prapare/ (Accessed June 24, 2021).

[16] Saunders B Sim J Kingstone T Baker S Waterfield J Bartlam B . Saturation in qualitative research: exploring its conceptualization and operationalization. Qual Quant. (2018) 52:1893–907. doi: 10.1007/s11135-017-0574-8, 29937585 PMC5993836

[17] Birt L Scott S Cavers D Campbell C Walter F. Member checking: a tool to enhance trustworthiness or merely a nod to validation? Qual Health Res. (2016) 26:1802–11. doi: 10.1177/1049732316654870, 27340178

[18] Morris ZS Wooding S Grant J. The answer is 17 years, what is the question: understanding time lags in translational research. J R Soc Med. (2011) 104:510–20. doi: 10.1258/jrsm.2011.110180, 22179294 PMC3241518

[19] National Center for Complementary and Integrative Health. *Whole person health: What you need to know 2021*. (2021). Available online at: https://www.nccih.nih.gov/health/whole-person-health-what-you-need-to-know.

[20] U.S. Department of Health and Human Services. *ICD-10-CM Official Guidelines for Coding and Reporting 2018*. (2018). Available online at: https://www.cms.gov/medicare/coding/icd10/downloads/2018-icd-10-cm-coding-guidelines.pdf.

[21] McQuistion K Stokes S Allard B Bhansali P Davidson A Hall M . Social determinants of health ICD-10 code use in inpatient pediatrics. Pediatrics. (2023) 152:319. doi: 10.1542/peds.2022-059319, 37431596

[22] Truong HP Luke AA Hammond G Wadhera RK Reidhead M Joynt Maddox KE. Utilization of social determinants of health ICD-10 Z-codes among hospitalized patients in the United States, 2016-2017. Med Care. (2020) 58:1037–43. doi: 10.1097/MLR.0000000000001418, 32925453 PMC7666017

[23] Guo Y Chen Z Xu K George TJ Wu Y Hogan W . International classification of diseases, tenth revision, clinical modification social determinants of health codes are poorly used in electronic health records. Medicine (Baltimore). (2020) 99:e23818. doi: 10.1097/MD.0000000000023818, 33350768 PMC7769291

[24] Hendricks-Sturrup RM Yankah SE Lu CY. Social determinants of health Z-code documentation practices in mental health settings: a scoping review. Health Aff Sch. (2024) 2:46. doi: 10.1093/haschl/qxae046, 38756172 PMC11050653

[25] Luke MJ Scribano PV. Z-codes: the first step in overcoming barriers to social determinants of health documentation. Pediatrics. (2023) 152:2205. doi: 10.1542/peds.2023-062205, 37431598

[26] Sullivan GA Gely Y Donaldson A Rangel M Gulack BC Shah AN. Use of international classification of diseases, tenth revision, clinical modification Z codes to identify social determinants of health among surgical patients. JAMA Surg. (2022) 157:735–7. doi: 10.1001/jamasurg.2022.1211, 35675058 PMC9178493

[27] Diaz A Pawlik T. Association of ICD-10 clinical modification codes for social determinants of health with surgical outcomes and hospital charges among Cancer patients. Ann Surg Oncol. (2024) 31:1171–7. doi: 10.1245/s10434-023-14501-4, 38006529

[28] Pollak YLE Lee JY Khalid SI Aquina CT Hayden DM Becerra AZ. Social determinants of health Z-codes and postoperative outcomes after colorectal surgery: a national population-based study. Am J Surg. (2022) 224:1301–7. doi: 10.1016/j.amjsurg.2022.06.012, 36031423

[29] Zaleta AK Miller MF Fortune EE Olson JS Rogers KP Hendershot K . CancerSupportSource(TM) -caregiver: development of a distress screening measure for cancer caregivers. Psychooncology. (2023) 32:418–28. doi: 10.1002/pon.6092, 36604371

[30] Chen HL Annie Kao TS Reuille KM Northouse L. FOCUS program: treating patients with Cancer and family caregivers as a unit of care. Clin J Oncol Nurs. (2021) 25:E17–e25. doi: 10.1188/21.CJON.E17-E25, 34019018

[31] Longacre ML Applebaum AJ Buzaglo JS Miller MF Golant M Rowland JH . Reducing informal caregiver burden in cancer: evidence-based programs in practice. Transl Behav Med. (2018) 8:145–55. doi: 10.1093/tbm/ibx028, 29385550 PMC6257028

[32] Dockham B Schafenacker A Yoon H Ronis DL Kershaw T Titler M . Implementation of a psychoeducational program for cancer survivors and family caregivers at a cancer support community affiliate: a pilot effectiveness study. Cancer Nurs. (2016) 39:169–80. doi: 10.1097/NCC.0000000000000311, 26496519

[33] Applebaum AJ Schofield E Kastrinos A Gebert R Behrens M Loschiavo M . A randomized controlled trial of a distress screening, consultation, and targeted referral system for family caregivers in oncologic care. Psychooncology. (2024) 33:e6301. doi: 10.1002/pon.6301, 38363002 PMC11250988

[34] Applebaum AJ. Caregivers clinic at memorial Sloan Kettering Cancer center. GSA 2021 annual scientific meeting. Phoenix: Innovation in Aging (2021).

[35] Administration for Community Living. *RAISE Act Family Caregiving Advisory Council, Advisory Council to Support Grandparents Raising Grandchildren*. 2022 National Strategy to support family caregivers. Administration for Community Living (2022).

[36] American Association of Retired Persons. *Supporting family caregivers providing complex care*. (2021). Available online at: https://www.aarp.org/pri/initiatives/supporting-family-caregivers-providing-complex-care/.

[37] Coleman EA. Family caregivers as partners in care transitions: the caregiver advise record and enable act. J Hosp Med. (2016) 11:883–5. doi: 10.1002/jhm.2637, 27378748

