# Supplementary material for: Social determinants of health in lung cancer surgery: perspectives from family caregivers, clinicians, and healthcare system administrators
Source: Front Public Health. 2026 Jan 15;13:1681825. doi: 10.3389/fpubh.2025.1681825 (PMC12852436; doi:10.3389/fpubh.2025.1681825)
Supplement: Supplementary file 1 [file Supplementary_file_1.docx]

Supplementary Material

# Appendix A. Family caregiver’s key informant interview guide

**Phase 1**: Share a video about the tail of two zip codes: <https://www.youtube.com/watch?v=Eu7d0BMRt0o>

- Have you ever heard of this concept that your zip code is more important than your genetic code?
- What were your impressions of the video? What resonated with your current experiences?
- Which town most represents your current living situation A-Town or B-ville?

*Neighborhood and Built Environment: As described in the video, where you live has a major impact on your health and quality of life.*

1. How would you describe the community that you live in? Describe the safety of your community, your water, street, open air space, pollution, and park availability, etc.
2. Has where you live affected your caregiving role? If yes, how?
3. If available, would you be interested in learning more about environmental exposures in your community? If yes, what kinds of information would you like to know?
4. Do you feel that environmental exposure information would be helpful in planning your healthcare?
5. Do you feel that environmental exposure information would be helpful in planning the healthcare/cancer care for your patient?

*Social and Community Context: We want to know how your relationships and interactions with your friends, co-workers and community impact your quality of life.*

1. How would you describe your experience of caring for the lung cancer patient?
2. In your opinion, what do families who go through a lung cancer diagnosis and treatment need most?
3. What did you need the most as a caregiver going through this process?
4. What type of social and community support did you need the most?
5. How has the lung cancer diagnosis changed your relationship with yourself?
6. How did this change impact your various roles (e.g., mother/wife, parent, etc.)?
7. How would you describe your social support network and has anything changed since becoming a family caregiver?
8. How are you accessing your social network to support your role as a caregiver? Provide specific examples of how?
9. As a caregiver, how have you been coping?
   1. how did you manage the stress that comes along with a serious illness like lung cancer?
10. Do you use or have you used religion or spirituality to support your role as a caregiver?
11. Describe your religious and/or spiritual background.
12. When did you use religion or spirituality in your caregiving journey?
13. How important was this usage in your caregiving journey?
    1. If no, how did you cope with the stressors of your caregiving role?

*Health care Access and Quality: Health care access services can depend on several socioeconomic factors. For example, 1 in 10 people do not have health insurance or access to the health care services they need. These questions are going to focus primarily on you.*

1. What are your views about the healthcare system during your role as a caregiver?
   1. How have your views regarding the health care system changed, if any (from your caregiving perspective?)
   2. How were you treated by the care team at City of Hope?
   3. That your care team sees you as a person, or only as a caregiver to carry out the required tasks?
2. How would you describe your health care access and the quality of resources you have? Is this similar to the patient.
3. Did your own health change during this time?
   1. How did your own health change during this time?
4. Did you engage with the healthcare system to support your health?
   1. Please describe your engagement with the healthcare system.
   2. How far away is your provider?
   3. Do you think this is a reasonable distance to travel for care?
5. Are there are any services that you need as a caregiver that you are not receiving?
   1. Describe the type of support you received during this time.
   2. What types of support did you need but did not receive?
   3. How would receiving these services improve your overall quality of life?

*Education: Research tells us people with higher levels of education live healthier and longer lives.*

1. What are your thoughts about this statement?
2. What is the highest level of education of any type that you have completed?
3. Do you think your educational background affected your caregiving role?
   1. How did your educational background affect your caregiving role?
4. Are there additional questions I should ask to determine how much education someone has?

*Economic Stability: This domain focuses on your economic mobility, food security, employment, and housing.*

1. Based on the definition I just read, do you have any unmet economic concerns that you need support with?
2. What are those concerns? Please provide examples related to your physical, spiritual, or psychological health, if appropriate.
3. How would meeting these concerns improve your overall quality of life?
4. Would addressing these concerns also improve the quality of life of your patient? Why or why not?
5. How could the care team have supported you during this time?
6. Has your economic condition affected your caregiving role?
7. How has your economic condition affected your caregiving role?
8. Has your caregiving role impacted your economic condition?
   1. How has your caregiving role impacted your economic condition?
9. In the last 12 months, have you worried whether your food would run out before you got money to buy more?
10. In the past 12 months have you felt, the food that you bought just didn’t last and you didn’t have money to get more?

*Before you go, please answer these last two questions to let us know if we missed any information about your journey.*

1. Do you feel that social determinants of health information, like the ones you shared with us today about your *environment, you social network including coping, education*, should be included as part of your treatment plan discussions with the patient’s treatment plan and discussed with the cancer care team?
2. How could this information be used to support you and your family?

That concludes our interview. Thank you so much for coming and sharing your thoughts and opinions with us. If you have additional information that you did not get to say during the interview, please feel free to email me at dkteteh@mdanderson.org.

# Appendix B. Provider and administrator’s key informant interview guide.

1. Please describe your role at ______________ (Name of institution).
   1. How many years have you been in this role?
   2. What do you enjoy the most about your position?
2. How do you describe the concept of social determinants of health (SDH)?
3. Does SDH have a place in healthcare delivery? Why or why not?
4. Do you think SDH related needs affect lung cancer patients? If yes, how?
5. Do you think SDH related needs affect family caregivers? If yes, how?
6. Does SDH have a place in lung cancer surgery? Why or why not?
7. How are SDH needs identified at your current institution?
   1. If SDH needs are identified, how is this information:
      1. Collected?
         1. When is this data collected?
         2. Is this data included in EHR records? Why or why not?
         3. How does the care team collect SDH data on patients within the EHR?
         4. Which SDH needs are collected in data collection efforts?
      2. Reviewed?
         1. Who should be keeping track of this information?
         2. Is there a space for EHR to facilitate this process?
      3. Acted upon?
         1. Does the care team ensure patients receive up-to-date referrals? Why or why not?
8. If SDH needs are not identified, should they? Why or why not?
   1. How should this information be __________?
      1. Collected?
         1. Which SDH needs should be included in data collection efforts?
         2. When should this data be collected?
         3. Should SDH data be included in EHR records? Why or why not?
      2. Reviewed?
         1. Who should be keeping track of this information?
         2. Is there a space for EHR to facilitate this process?
      3. Acted upon?
   2. How do care team members want to ___________ on patients’ SDH needs within the EHR?
      1. Collect?
      2. Review?
      3. … and act on data?
   3. How can the care teams ensure that patients receive up-to-date referrals?
9. Any final comments or thoughts you would like to share before we conclude today’s discussion?

That concludes our discussion for today. Thank you so much for speaking with us today. If you have additional information that you did not get to say during today’s discussion, please feel free to email me at dkteteh@mdanderson.org.

# Appendix C. Family caregivers’ themes by social determinants of health domains and example quotes.

***Theme 1: Knowledge about SDOH Factors and Integration of SDOH Information into Healthcare.***

| **Sub-Themes** | **Example Quotes** |
| --- | --- |
| Acknowledgment of social determinants of health impact on health outcomes | *“...there's so many factors and kind of aspects of life for the patient, the caregivers, everybody, that is a factor to the outcome. So yeah, I think having that information about access and support systems is more important than I kind of gave it credit for before going through this.”* |
|  | *“Well, knowing there's someone there for you when you need it and someone who cares. And can just step right in and help you, I mean. I just can't explain it. I feel for someone who doesn't have family of any kind to help them.”* |
|  | *“It was a little shocking to me, maybe more so for myself that I didn't piece that together to think that it could impact healthcare too. But I just never thought of that as I guess part of the umbrella that all of those things can impact you know like I said my dominant thought was about education. But yeah, overall health that, that was a little surprising to me”* |
|  | *“You know, because the healthier environment you live in and the healthier choices you have, you know, makes it convenient. You know, like for, like farmers market or healthy food compared to, you know, like the others ZIP code, which is, you know, just like fast food. Not places where you really want to go out and take a walk in the park, you know, just mainly you wanted to stay inside because you're some people could be, you know, feared for their safety by going out. Instead of like in the you know like say number one was a healthy, number two was not the healthy so but like number one healthy where you can you don't have a problem walking outside and walking around and just enjoying yourself compared to you know being cooped up in your house you know, afraid of going out.”* |
| Integration of SDOH information into healthcare | *“I think it's important to her team should know that she has adequate support, for when they're not, when she’s not in her in their immediate care”* |
|  | *“You know, I think for some people, especially if the professionals knew where their background was and some of those questions that they asked initially on, if they were just like a getting to know you, it could definitely impact how the doctors might put some of that information to the families that are going through this for sure… So, I can see how that directly impacts and I know for a fact my mom, you know, if she didn't have somebody like me advocating on her behalf, she might have just waited. Because they think that the doctors are gonna do what they're supposed to, you know. So I do think it's important.”* |
|  | *“That's hard to say because I'm thinking that when the diagnosis is given, that you're in a situation where there's not much you can change. I mean, you're at the economic situation and you're in the, you know, neighborhood you're in, you've got the family that you have. You know, it's like you can't really add or make changes at that point.”* |

**Economic Stability (Theme 2): *Caregiving burden is minimized by economic mobility opportunities*.**

| **Sub-Themes** | ***Example Quotes*** |
| --- | --- |
| None or no immediate economic stability concerns | *“No. Well I am 68. So, I'm getting close to retirement, very close. There [are] some concerns of, well, if I'm gonna be able to live comfortably on retirement. I haven't done that full analysis yet. At this particular point, my employment is still good.”* |
|  | *“Yeah, there are a large home was. Yes, it was only about half a mile away. Yeah, it was lovely. We had a nice view at this of the San Gabriel Valley and it was. It was great when we were younger, but a big home takes a lot of work and expense and so we downsized, and we love where we are. Our children are not happy about it because there's stairs again, but it's hard to find a place in this area without two stories.”* |
|  | *“As far as employment of course, we were short staffed as usual because of this pandemic. Can't find help that is decent enough to do what we do. But you know, we work with all our employees as much as we can. As far as, we made it thru COVID. We stayed open. We had you know we had savings and stuff, we were okay, you know, we didn’t suffer. But you know, other people did. Yeah. And then as far as, nutritional and all that, we were fine, I mean, food was not an issue here in town.”* |
| Caregiving burden experienced by the “sandwich generation” reduced by social support | *“I feel really fortunate that I got to really just focus on caregiving. I work full time. But my job, you know, knew the date of the surgery. So, I was able to take the time off of work without any kind of thing hanging over in that regard. Or like I said, my husband my we have two kids and my husband kind of held down their schedule and everything they needed back home so I was able to go stay with my mom for the time that she needed me.”* |
| Caregiving burden impact by employment status, income, and healthcare access | *“I mean you know when you don't have to worry about other external matters, it's easier to focus on what you should be focusing on the caregiving aspect. Also, it was when I was transitioning to retirement when she was having the surgery. And so, I had a little more time to help out. I wasn't stressed with work and with other commitments, and so I could focus just on her and then, you know, the way that [study site] prepared us for that was really good because they give us lots of information. And knowledge is power.”* |
|  | *“No, everything's good. Once again, I think there's advantage being older, being, you know, retired or closer to retirement, like when [patient name deleted] had it. Because I'm thinking back if this had happened when we had our three kids when they were smaller. It would be a different situation I mean I wouldn't be able to just to work or concentrate on helping [patient name deleted]. I have three other kids to take care of.”* |
|  | *“…the person that gives my haircuts. She has cancer and she's telling me the treatments that she's getting and I'm thinking, oh my gosh, this is like, no. And I tell her, but because of probably where she's at financially and just not having the wherewithal to actually help. Because I've asked her, hey, want me [to help] you out? You know, like, talk to these guys. But she says no. And I'm like, wow, this is not the best treatment, but it's just that where she's at in her, you know, economic status. But also, just not having the experience or having another like a patient advocate trying to help her that she's getting, I'm thinking like marginal care. Whereas my son had a liver transplant at UCLA, because his wife was able to navigate the system and find the best doctor for that for what he had.”* |

**Education Access and Quality (Theme 3): *Degree attainment, supplemental education, and lived experience impact quality of life of lung cancer surgery caregivers.***

| **Sub-Themes** | **Example Quotes** |
| --- | --- |
| Salutatory impact of education on quality of life | *“Oh, I mean just makes sense. People with education, easy to take better care of themselves. You should pay more attention to their environment that they live in. It just goes back to that video, people with education tend to live in a better environment. Doesn't turn, you know, probably. Live longer. We used to live right off the 5 freeway in Burbank and within half a mile and I knew it wasn't good. It was an okay neighborhood, but I didn't like being that close to the freeway, you know.”* |
|  | *“I do think that [it] is impactful. I had [several] years of teaching state preschool and for state preschool, it's an income qualified program. So, a lot of our families were low income and subsequently there's a relationship with lower educational levels. And so, just in healthy eating habits and making sure that children are moving, it was surprising to me early on in teaching how much parents did it know. And that those everyday choices were impacting the health of their kids and that setting those habits at a young age and preschool, how it can impact their lives long term.”* |
|  | *“I think it definitely has got to be a huge factor in our health, just because we have, you know, with education, you have more opportunities I think and you know, access to the information to make good health care decisions. Yeah, well, I work in healthcare, so I felt like that was helpful. I don't work… I'm below the waist. I'm an OBGYN, so lungs are a whole different ball game. But yeah, just having that you know, kind of basic medical knowledge I think was really helpful understanding the healthcare system. It kind of helps you navigate right, when you're on the other side.”* |
| Supplemental education to support caregiving role | *“I think I really appreciated all the information. So, I think it's important obviously to kind of understand what someone’s baseline you know understanding is of the diagnosis or of the intervention. But I really appreciated that even though my mom’s healthcare team knew that my husband and I had a medical background. They still were very...they took the time to kind of explain all the details. They didn't assume because I, you know, that I don't know why. So, it was good that they didn't assume. And so, I got. I felt like we got a really good base of knowledge. But yeah, I think it's always important to meet someone where they're kind of base is and then go from there.”* |
|  | *“Yeah, I agree. Once again, knowledge is power. I mean someone that you know, I've been fortunate to be exposed to a lot of things. I've got an MBA, and you know; I worked in a medical field. Like I said 35 years and such. And just having that, this not being a foreign environment to me, I think is a big help. I think someone who has been in hospital before this type of thing, it could be overwhelming. But you know knowledge is power having that information available and having that experience was a big help. And City of Hope, you know, put my mind at ease that that was big. It was huge.”* |
|  | *“It's not only Higher education is also an experience. Because, you know, I went through what my dad had to go through. And being the primary care. So that enabled me to help my sister. So I don't know if other peoples are, I wouldn't say fortunate. I don't know if you could say it's fortunate to have that experience cause it was kind of rough.”* |
| Non-educational predictors of quality of life | *“It just depends on the individual itself. I mean, I know a lot of people that don't have any education. They're like in perfect healthy condition. Um a good friend of mine, no education. I mean, well, you know, high school education, but 82 years old and great health, you know, very healthy. Then just depends on you know the individual itself, you know. So, I mean it does, you know, education more or less it does help but street smarts are sometimes way better than education, you know, just depending on each individual itself.”* |
|  | *“Well, if a person has more education, they may be aware. They maybe aware that they need to take better care of themselves. They may know how to do that. Because of their higher education might tell them how to take better care of themselves. But I think a person with a lower educational level should have been able to acquire the knowledge [of] how to take care of themselves. How to look after themselves. I don't think it has to do with the level of your education. Bottom line.”* |

**Healthcare Access and Quality (Theme 4): *Navigating the healthcare and cancer care delivery systems.***

| **Sub-Themes** | **Example Quotes** |
| --- | --- |
| Switching insurance to have more choice in cancer care | *“Her insurance determines where she can go.... Boy, we had to move heaven and earth.... I went to the Internet, started searching everything I could find on trying to get her down there.”* |
| Bypassing referral systems to receive timely care | *“We were pretty much to ourselves here....because it all happened really fast with us. She got diagnosed and then we tried a different route and went straight to [location of study site]. As opposed to going through the whole referral system because we knew time was of the essence. So rather than going through her general than going through this one referral, all knowing that takes months and months, we skipped all that.”* |
|  | *“...when she was first diagnosed we reached out to [study site] because her primary never got back to us with the test results. I had to call the hospital where she had the test done, and luckily she was able to confirm. But it was because there was a delay in hearing from her healthcare provider....she would have got a referral from her doctor, which we were waiting forever. And so, calling [study site], letting them know her story, they were amazing because, you know, it wasn't like we need to hear from your primary. It was okay. Here's an appointment. Let us know who she's seeing, where she had her test. We'll pull that information for you and you know.”* |
| Empowerment, advocacy, and persistence | *“....it's important to be an advocate to have either for yourself, for a patient, a patient advocate.”*  *“It's still, you know, people have options, but you also have to use your own wherewithal to get the most out of those options and not just get stuck. And I can see if someone doesn't have any options and they'll you know, they're gonna be treated in different ways.”*  *“But I think maybe empowering patients to know that they are in charge of their own healthcare, and they have options.”* |
|  | *“And I was very persistent because a lot of them couldn't get her in for, like, a month to six weeks after. I'm like, you got to be kidding me. You know? I'm like, we're in the hospital. She got it done right then, you know, well Sir, we're really busy. And I said, well, you need to put me on, you know, a cancellation list or something. And so, I would actually call them all up, like twice a day. And I was going to be very persistent. And you know, finally there was a cancellation at one of the locations and we were able to get her in within like 3 days....I don't mean to be a pain, but you know, we need to get her in there because we don't know if it's just the isolated situation or if it's metastasized and it needs to be taken care of right away.”* |
| Accessing quality information on lung cancer | *“So much of what we get is off the Internet. Which it has, as we all know, good and bad. The truth of it is good. The part that's not accurate to your situation is devastating. You know, like we were reading about her one cancer was like, Oh my God. We come to find out, she had this type that was very treatable so. A big part would be, what people need is education and support and knowledge. Especially those are going through the hard times of it.”* |

**Neighborhood and Built Environment (Theme 5): *Proximity to healthcare facilities and neighborhood environmental factors impact caregiving role.***

| **Sub-Themes** | **Example Quotes** |
| --- | --- |
| Caregiver burden of transportation & distance to medical care | ***“****Granted, it was a long distance to the treatment. I went down with her on most of her treatments. And then they moved it ... closer. But of course I went with her. Sat in the parking lot. Well, originally when she had her operation stuff, we had to go to [study site], which was almost two and a half, three hours. And then we moved to treatments to [study site], which is an hour 20 minutes. And those treatments took about three to four hours.”* |
|  | *“We were always having a hard time ... because we traveled down to [study site]. We don't live near there. So, it takes us about 2 hours to get there. So, it's a lot of drive time on her. I don't think some people realized that we were driving you know, to her appointments and I just wish people would kind of maybe ... recognize what people, what their situations are coming into this. So, she's having this procedure. How do I manage that with my mom in the car? Because I know there were times where my dad, he would take us down there and I said, dad, she needs to go to the bathroom. We have to stop, or we said, ‘Mom, you need to take this in the car with you when you go down there because I know you get thirsty and it's gonna help with your electrolyte.’ A little bit, people telling us more, how to travel down there with somebody that has cancer and going through chemo because she would get nauseous in the car coming back.”* |
| Concern about environmental exposure causing cancer | *“I think it's about his exposure to the area where he worked too long and without a mask, without protecting himself. And then it could be causing his lung cancer.”* |
|  | *“Most likely her environment. My sisters... they're all very concerned about how my mom got this cancer. When she's never been a smoker in her life.”* |
|  | *“There's a factory there that just produces a lot of pollution and ... that's in that county and the health over there is not that great.”* |
|  | *“I was thinking about my mom all the time ... like, how did she get cancer? How did this happen to her when they did all the testing? ... and they just kind of narrowed it down to the environment she lived in, which doesn't surprise me living here.”* |
| Importance of neighborhood and built environment for ease of caregiving | *“So, our neighborhood, we have a lot of... healthy options and resources really close by, like farmers markets and grocery stores full of produce and fresh options... We're pretty close to the beach, so we have access to a lot of public outdoor spaces. And it's a pretty safe community....I'm sure it has, in a lot of ways, made it easier to be available to provide that care... And living in the community I live in, I feel really fortunate that I have easy access to get out and go for a run or, you know, get outside to kind of help clear my mental health as well.”* |
|  | *“Where we live, it's a master plan community... so everything was built out with the parks in mind, with the wide streets, the neighborhoods, the churches, the family... We have mountains close by. We can go hiking and such. And so we're close to a lot of recreational activities, and the city itself was built in such a way so that it would be conducive to all those things. So people wouldn't have to drive far away. Their jobs are right here in the city. And so you don't have to get on the freeway and waste 45 minutes of your life every morning and coming back an hour with traffic. You can work here close by... When you don't have to worry about other external matters, it's easier to focus on what you should be focusing on the caregiving aspect.”* |

# Appendix D. Providers and administrators’ themes on integration of SDOH assessments in healthcare delivery and example quotes.

**Theme 1: *Importance of SDOH integration into healthcare delivery models for patients.***

| **Sub-Themes** | **Example quotes** |
| --- | --- |
| Healthcare system reckoning on the reality of inequity and fragmentation of care | *“The question is like do we in health care recognize that reality right? Like there's no question that social determinants of health influence life and health. The question is just to what degree are we totally ignoring that reality?” - Administrator* |
|  | *“I mean it's such a huge, huge problem that you get. We're getting out into the whole health care system and our whole health care system is so broken...they were very clear that their goal was health care for everybody, for free....but with the financial issues and stuff. And that means that people who don't have good health care in primary care, they're not going to get to us.” - Provider* |
| SDOH models of care that are not entirely disease focused | *“I would say it's about interdisciplinary care. So, changing cancer care to be interdisciplinary is like adding a whole new set of tools, because now we have a number of different lenses, a number of different people looking at the same patients. But now what we have is a lot of different lenses, a lot of different people with different kinds of tools who look at this patient differently but plan the care differently.” - Administrator* |
|  | *“It’s really easy for providers to get caught up in the clinical side of caring for a patient that they forget that there is a huge social side of caring for patients. And that a patient’s mental state has a huge impact in their physical state. Too many providers I think are focusing on the medical management side of things and then the other side of the coin, which can greatly again impact the physical being, get downplayed or forgotten.” - Provider* |
| Sustainability of SDOH-driven models of care | *“In order to address those needs, and so they're kind of putting in their heels. And so, my job really has been to talk to them and to find out what exactly they need in order to really address the needs of those patients. And then advocate for the different teams and create conversations with various leaders so that we can secure a financial infrastructure. Because if they're serious about addressing the needs of vulnerable and the social determinants of health of our patients, then we have to invest the dollars in the infrastructure in order to make sure that we have the people there necessary to follow through on the needs that we assess in the patients.” - Administrator* |
| Interconnectedness of SDOH and quality of life | “*I think you know there are too many studies that have shown that these factors impact outcomes, right? And quality of life and even survival, right? So to not be privy to them, really sort of cuts is that their needs are like if we didn't know that a patient had diabetes or if we didn't know that their kidney’s didn’t work.” - Provider* |
|  | *“Once you get the disease itself and you need much more, the delivery of care becomes so much more complex and the disparities become enormous and determinants of health become very, very important.” - Provider* |
|  | *“When we say social determinants of health, it's not just we the researchers pick out. Oh, I'm gonna use the variable of gender. You know, I'm gonna use the variable of age and then try to explain the whole world based on my, you know, independent variables. Instead, it's social determinants of health are about me entering the world of the other and listening intently to what is your worldview, what is your life experience and how does that experience influence how you then have cancer and cancer treatment, right?” - Administrator* |

**Theme 2: *Interdisciplinary approaches to collection of SDOH data in healthcare delivery for patients.***

| **Sub-Themes** | **Example quotes** |
| --- | --- |
| Collection of SDOH data in healthcare delivery | *“We should have a system in place that says that before the patient, you know, leaves the hospital from the surgery that a social professional is social worker, psychologist.” - Administrator* |
|  | *“So I do think that nurses are sort of in the trenches, frontline generalist level and then we need to have these support services for when we do recognize that this is a patient that has significant social determinants of health that are gonna influence their outcome of what happens after the surgery” - Administrator* |
|  | *“I personally believe that quality cancer care that every patient should be seen by a social worker” - Administrator* |
|  | *“Well, I mean I again, I think algorithmic care is there is the right way to go. I think obviously that the surgeon and the and his health care team, the nurse practitioner, the physician assistant, they all have to know that information.” - Provider* |
|  | *“Because knowing who this person is and about a lot of social determinants of health should inform how I do my patient teaching. Right? So shared information and a well-integrated plan of care is what people deserve, right?” - Administrator* |
|  | *“I think I mean it probably has to be a shared responsibility which is I know difficult because without identifying ownership two things can happen. Either everybody is addressing it, and everybody is addressing it just a little bit differently. So, it becomes very fragmented. Or people assume, well, that you know, physician says, will the nurse practitioner will do it, and then the nurse practitioner says, well, you know the clinic nurse will do it. And then the clinic nurses, while social worker will do it. And then nobody does it. So that is something I think that would have to be worked out. Like who owns that information and that assessment?” - Provider* |
|  | *“If we all just go and talk to the patient and then we all just go back to our own worlds like, have we accomplished anything? No, only when this interdisciplinary assessment makes it to the chart and then begins to impact.” - Administrator* |
| Utilization of Electronic Health Records (EHRs) | *“Absolutely so in essence, so our goal is to ask a couple of questions about financial security, housing security, food security or insecurity and transportation. And so, there are questions already in Epic, and so we have a working group that wants to pull these questions out so that every individual who comes into the City of Hope will be asked these questions by the care team, whether it's the nursing or the medical assistance like as a part of hey, do you smoke? How much have you smoked? When did you stop? Oh, by the way, you know, do you have a home or your home?” - Provider* |
|  | *“I know that there are questions in Epic because I see them like in intake. But I don't know how accurately they're actually completed, like the nurses have so much to do, like in terms of charting and asking.” - Provider* |
|  | *“Now they're working on a workflow. Well, the nurses when they're doing the in rooming before an appointment, they ask the questions of the patients that are along the domains of social determinants and then they go back and enter it into their computers so that when the doctor goes into the room. they'll be able to see if things were identified in the different domains.” - Administrator* |
|  | *“I guess that's the utopia of the electronic medical record is that going, moving downstream, it's going to get progressively better and you know we're going to continuously refine what we do the way we take care of people.” - Provider* |

**Theme 3: *The role of Family Caregivers (FCGs) in cancer care delivery in the context of SDOH.***

| **Sub-Themes** | **Example quotes** |
| --- | --- |
| Essential role of family caregivers in cancer care delivery | *“I mean, yes, family caregivers should be part of the team, but it almost like minimizes to me that, like, no, they are the team when it comes to home care. They are all things, right.” - Administrator* |
|  | *“I don't think it's possible to provide quality patient care for a serious illness like lung cancer and not do a good social assessment and not know something about the family caregiver, right? Like, it's impossible. You can't do it.” - Administrator* |
|  | *“I tell patients all the time or patients’ families when they come with a concern or something. I tell them that you're just as much as part of this care team than the patient and us.” - Provider* |
|  | *“I do think that there is not enough you know, in general, involvement of family caregivers in you know everything you know just in terms of like leveraging the family, caregivers and really understanding what the situation is. I think a lot of people ask those questions, but there's probably a lot of people who don't. Providers who don't, so I think, maybe like educating providers is important about just asking questions about the support system and like just in general the social determinants of health where they're coming from. Getting a picture of, you know what life is like for this patient.” - Provider* |
|  | *“But we also have to remember the family caregiver is a person, too. They exist beyond their caregiving role. And so I think that failure to really, you know, acknowledge and understand and approach the family caregiver as a unique person, not just as a you know, add to the patient is a gap, right?” - Administrator* |
|  | *“And so most clinicians, I don't think they really understand, you know what that looks like at home that this family member is really providing 24 hour day nursing care. And a lot of times they're providing, you know, 24-hour day intensive care because you know, they're doing it all. They're managing symptoms and medications and appointments and mood and food. And, you know, it they're doing. It's an enormous 24-hour day responsibility.” - Administrator* |
|  | *“Well, I think we need to broaden our perspective on who a caregiver is. And I also think that we need to discover the level of coping that the patient has, whether or not they need paternalistic care, and whether or not they want to be seen as a partner in their care. Because that also plays into, you know, the role that that the caregiver will give. And I think a lot of times, and especially in our fragmented society, that caregivers aren't necessarily family members. Sometimes they're just really close friends. Sometimes their coworkers and their neighbors, you know. So, we need to kind of think about the context and how they build that out.” - Administrator* |
| Integration of SDOH information into cancer care delivery | *“But if we're thinking talking about income, education, we talk about neighborhood context, social contact, and we're talking about thread like pure social determinants. I think you get most of what you need, like 90% by asking the patient about theirs.” - Provider* |
|  | *“I think it's one of those like would be nice, but in practical speaking, you know I'm not sure I feel like there's a lot of barriers to doing that.” - Provider* |
|  | *“Well, for one you know, the caregiver is not the patient. So just like the actual documenting someone other than the patient like, how do you do that? Do you document it in the patient’s chart? And I think you know that caregivers may not be present, you know for the meeting. And then also like what level of detail like anytime you start to collect information like this, you have to think like okay, am I collecting this for clinical use for research use because like for clinical use, you have to like you know, be a little bit more narrowing the scope of what you're asking usually, so you're finding that balance between collecting enough information and not overwhelming the people who are collecting the information. And so, I feel like that's even more maybe the case with the caregivers.” - Provider* |
|  | *“We've seen caregivers die and I think again that the research, the, the use of the electronic health record to conduct research on the needs of caregivers is something that's brilliant” - Provider* |

**Social and Community Context (Theme 6): A*vailability of social support and caregiving coping strategies.***

| **Sub-Themes** | **Example Quotes** |
| --- | --- |
| Availability of social support | *“And so yeah, I think I just leaned on my family, my brother and sister and mom. We just kind of tried to stay really open in our communications with what we were worried about or what, you know.”* |
|  | *“I felt like me and my younger sister grew closer for sure, cause it's just me and my little sister that live here on over at the coast, so she wasn't here as much. And then my third sister, unfortunately she is not talking to my parents.”* |
|  | *“It was just me and myself because with COVID everything going around we didn't want anybody coming over to the house and we just basically, you know, stayed away from everybody.”* |
| Shifts to caregiver roles and responsibilities | *“And then I helped her to change her bandages, and then I helped her with flushing that stuff that she has to take out that builds up. And flush it down the toilet and then clean the site with some alcohol and stuff. Get her some food and stuff. It's basically trying to comfort her.”* |
|  | *“So, I find myself doing a lot more in terms of general household things than normal.  [I do] such things as laundry, house cleaning and those types of things. She doesn't have the stamina or strength to do the things that she used to do.”* |
| Caregiver coping strategies | *“Running makes me feel like I use it for my mental health for a million different reasons, and I always feel better after a nice long run outside, so I've been using that quite a bit to cope.”* |
|  | *“I took up the piano. I started playing the piano, and I found that kind of therapeutic.”* |
|  | *“I increased my walking. I wanted to start doing some, like, more vigorous exercise, but it was just kind of too much at the time. So, you know, I've been walking a whole lot and, in the afternoons, we walked probably, you know, 45 minutes. And then since I've been back at work and in August, I've been walking in the mornings and in the evenings to kind of help my dog with his anxiety and help me relieve any stress that I might have. But a lot of walking.”* |
|  | *“Like I'm going to the ping pong game daily to the Senior Center. I talk to the people; I eat out with my sister and my father and I'm happy”* |
|  | *“I'm an artist and I finally have said a few weeks ago, I said I have to sit down and do some cards because I sell cards and there's a lovely store in another town near us. And I said I just have to sit down and do it, he says. Do it and I feel guilty I shouldn't, but I said, just pretend I've gone to work today and I go up there and I shut the door and I paint and that is that is a great stress reliever…”* |
| Use of religion/spirituality as coping strategy | *“…the only thing is I believe in the Buddha. But sometimes it's I pray a little bit but not like a deeply religion for praying. And I just pray actually talked to my mom. My mom already [passed]. So, I'll just [say] Mommy help me and support me and then let me go through all this. Yeah, that's why my prayers about it.”* |
|  | *“But me and my mom have gone to church every Sunday. Together we go every Sunday”* |
|  | *“For me, I wake up in the morning with a prayer. I do my meditation in the morning. So, the first thing I do before I get up and it's the last thing I do at night. Even during the day, you know. I don't let my mind ever go there. I only let my mind go to one place, and that's to my creator. I don't let it go anywhere else. I try to stay steadfast in that it's keeping going. “* |
|  | *Other than you know making a lot of prayers. You know no more than I usually would. You know, it's not like I turn into the religion you know for answers or something. But you know, I just use that as support just as a mental or moral support knowing that you know you pray hard enough, you're gonna get through something. You know whether good or bad, but it's going to help you get through it.* |
|  | *“Oh I believe in God and I talked to God a lot and he answers me and I feel very secure with my relationship with God. Yes, I don't go to church often enough, but I feel I have a I communicate with him and I have my prayers. That's my own way of dealing with it.”* |
|  | *“But yeah, in my mind spirituality has a lot to do with Peace of Mind and calmness”* |
|  | *“I prayed a lot.”* |
